# Delirium and cognitive assessment in national hip fracture registries: a scoping review

**DOI:** 10.1007/s41999-025-01246-4

**Published:** 2025-06-06

**Authors:** Niamh A. Merriman, Rose S. Penfold, Mary E. Walsh, Eithne Sexton, Louise Brent, Pamela Hickey, Tara Coughlan, Cristina Ojeda-Thies, Antony Johansen, Andrew J. Hall, Alasdair M. J. MacLullich, Niamh O’Regan, Catherine Blake

**Affiliations:** 1 School of Public Health, Physiotherapy and Sports Science, University College Dublin, Dublin, Ireland; 2 Ageing and Health, Usher Institute, University of Edinburgh, Edinburgh, Scotland UK; 3 School of Pharmacy and Biomolecular Science, Royal College of Surgeons in Ireland University of Medicine and Health Sciences, Dublin, Ireland; 4 School of Population Health, Royal College of Surgeons in Ireland University of Medicine and Health Sciences, Dublin, Ireland; 5 National Office of Clinical Audit, Dublin, Ireland; 6 Discipline of Medical Gerontology, School of Medicine, Trinity College Dublin, Dublin, Ireland; 7 Department of Age-Related Healthcare, Tallaght University Hospital, Dublin, Ireland; 8 Department of Traumatology and Orthopaedic Surgery, Hospital Universitario 12 de Octubre, Madrid, Spain; 9 Department of Surgery, School of Medicine, Complutense University of Madrid, Madrid, Spain; 10 School of Medicine, Cardiff University and University Hospital of Wales, Cardiff, Wales UK; 11 National Hip Fracture Database (NHFD), Falls and Fragility Fracture Audit Programme, Royal College of Physicians, London, UK; 12 School of Medicine, University of St. Andrews, St. Andrews, Scotland UK; 13 Waterford Integrated Care for Older People, Department of Geriatric Medicine, University Hospital Waterford, Waterford, Ireland

**Keywords:** Scoping review, Hip fracture, Audit, Registries, Cognition, Delirium

## Abstract

**Purpose:**

Delirium and cognitive impairment are common in older adults with hip fracture and are associated with adverse patient outcomes. The Fragility Fracture Network recommends that national hip fracture registries (HFRs) include a measure of cognitive status. However, inconsistency in recording of delirium and cognitive assessment data hinders international comparison and may reduce care quality. This scoping review aims to identify delirium and cognitive assessment data items collected by national HFRs and the associated key data reported in the latest annual reports.

**Methods:**

We searched three databases (Medline Ovid; Embase; CINAHL EBSCOHost) from inception to 18 November 2024 and relevant organisational websites. Two authors independently assessed titles, abstracts, and full texts for eligibility. Prespecified data items were extracted from identified eligible HFRs. Descriptive analysis was used to summarise findings.

**Results:**

Twenty-two eligible HFRs were identified. Of these, 14 (64%) collected delirium assessment data, 18 (82%) collected cognitive assessment data, while only one registry collected neither. There was heterogeneity in delirium and cognitive assessment tools, though seven (50%) HFRs recommended using the 4AT. Delirium assessment completion rates were 47.7–95.9% pre-operatively and 34.2–95.9% post-operatively, while positive delirium score rates were 14.7–22% pre-operatively and 5–42% post-operatively. Cognitive assessment tool completion and positive score rates were 58.5–100% and 9.9–52.4%, respectively.

**Conclusion:**

Most HFRs incorporate delirium and cognitive assessment data items, but there is heterogeneity in tools and methods. Improving the consistency of data collection across HFRs can improve comparability and patient care.

**Supplementary Information:**

The online version contains supplementary material available at 10.1007/s41999-025-01246-4.

## Key summary points

**Aim:**

This scoping review aims to identify delirium and cognitive assessment data items that are currently collected by national hip fracture registries (HFRs), to identify registry guidance for the administration of delirium and cognitive assessment tools across HFRs, and to report outcomes of these data items in the most recent annual reports of identified HFRs.

**Findings:**

Of 22 eligible HFRs, 14 (64%) collected delirium assessment data, and 18 (82%) collected cognitive assessment data. There was heterogeneity in recommended delirium and cognitive assessment tools (although 50% recommended the 4AT), cut-off scores, and tool completion and positive score rates.

**Message:**

While most identified HFRs recommended delirium and/or cognitive assessment, there was considerable variation in methods of assessment and documentation. Greater standardisation in data items and their collection could improve international comparability and patient care.

**Supplementary Information:**

The online version contains supplementary material available at 10.1007/s41999-025-01246-4.

## Introduction

Delirium is an acute and fluctuating neurocognitive disorder (NCD), characterised by disturbed consciousness, cognition, and attention [1]. Dementia is a major NCD marked by a deterioration in cognitive function that exceeds the typical effects of biological ageing and impacts a person’s ability to live independently [2]. Mild cognitive impairment is an NCD, characterised by a disruption to certain cognitive functions, such as memory, while maintaining independence in daily activities [3, 4]. Cognitive impairment is commonly referred to in research studies as a decline in cognitive function detected through positive test results, which may stem from various causes, including delirium, dementia or other neuropsychiatric conditions. These NCDs are common in older adults with hip fracture and are associated with adverse outcomes, such as decreased mobility, extended hospital stays, in-hospital mortality, and increased care needs after discharge [5, 6]. Up to 24% of patients with hip fracture have a pre-fracture dementia diagnosis, and 42% have cognitive impairment identified through cognitive assessment [7, 8]. As many as 21% of older adults with hip fracture have delirium at the time of initial presentation [9] and 10–51% develop delirium post-operatively [10].

Hip fracture registries (HFRs) facilitate identification of specific care processes and management strategies that are associated with better patient outcomes. At the national level, they provide a benchmark for hip fracture care against best practice clinical standards for acute care, rehabilitation, and secondary fracture prevention [11]. However, variability exists in the clinical standards set by national HFRs, as well as in the tools and timeframes used to measure outcomes. To enhance comparability among national registries and support the creation of new registries, a minimum common dataset (MCD) was first introduced by the Fragility Fracture Network (FFN) in 2014 [12]. It was later reviewed in 2022 by the FFN Hip Fracture Audit Special Interest Group (SIG), which includes appointed representatives from established national hip fracture programmes, international FFN members, and senior FFN administrators [12, 13]. The MCD represents the essential dataset that new national HFRs should strive to collect. It comprises 22 core questions and 12 optional fields, which can be utilised based on the healthcare system structure of each country [12]. A 2023 review of the comparability of HFRs using the MCD highlighted pre-fracture cognitive assessment at admission as a key area for enhancing compatibility across registries [14], with only 65% of included registries evaluating cognitive status. Additionally, the methods of assessment varied, ranging from standardised tools to basic documentation of a suspected history of dementia.

Detecting delirium in people with hip fracture is a critical aspect of care quality [14]. Early delirium identification is essential for many reasons, including identifying and treating causes, reducing risk of complications, informing prognostication, and enhancing communication with patients and carers [15]. Many national clinical guidelines and care standards advocate for routine delirium assessment, a fundamental component of acute geriatric care [16–20]. However, there is no international consensus on assessment methods or a recommended assessment tool. The impact of cognitive impairment and pre- and post-operative delirium on hip fracture outcomes require further research, which could be accelerated by greater consistency in definitions and assessment tools across HFRs [14, 21].

This scoping review aimed to identify delirium and cognitive assessment data items that are currently collected by national HFRs, to identify registry guidance for the administration of delirium and cognitive assessment tools across HFRs, and to report outcomes of these data items across the most recent annual reports of identified HFRs.

## Methodology

This scoping review followed the Joanna Briggs Institute (JBI) methodology for conducting scoping reviews [22, 23] and JBI guidance for engagement with knowledge users [24]. Findings are reported in accordance with the Preferred Reporting Items for Systematic reviews and Meta-Analysis extension for Scoping Reviews (PRISMA-ScR) reporting guidelines [25], as per protocol [26], published on 28 Oct 2024. The completed PRISMA-ScR checklist is available in Supplementary Table S1.

### Protocol deviations

Large-scale non-national HFRs were deemed eligible for inclusion if they reported a representative volume of registered hip fracture cases per annum. National registries under development were deemed eligible to ascertain whether they intended to collect outcomes relating to delirium and cognition.

### Eligibility criteria

Eligibility criteria followed the PCC framework (Population, Concept, and Context) for scoping reviews [23]. Table 1 outlines the inclusion and exclusion criteria.

**Table 1:** Eligibility criteria

*Inclusion criteria*
Population
People with hip fracture, defined as a break or fracture in the upper portion of the femur where the bone meets the pelvis [27]
Concept
National hip fracture registries or large-scale non-national hip fracture registries with a representative volume of included hip fracture cases that collect data for the purpose of monitoring and improving the quality of hip fracture care
Registries with continuous data collection that were in operation in 2024
Emerging national registries with established data dictionaries
Context
The hip fracture registry was considered as being at country level if it was reported as the accepted country-wide structure for data collection, or if it included the country’s name or the word ‘national’ in the title
Large-scale non-national hip fracture registries reporting a representative volume of registered hip fracture cases per annum
*Exclusion criteria*
Non-hip fracture populations
Regional or single healthcare system hip fracture registries

### Evidence sources

A comprehensive search strategy was developed, and three databases were searched (MEDLINE Ovid; Embase Elsevier; CINAHL EBSCOHost) from inception to 15 November 2023 (updated 18 November 2024). No date restrictions were applied and only abstracts with links to full texts were included. See Supplementary Table S2 for the full search strategy. Eligible HFRs were identified from peer-reviewed articles that signposted to a relevant registry or registries. Relevant websites, such as the FFN and organisational websites of the identified HFRs were searched. Citation lists of previous reviews and included studies were hand-searched. Chairs of national HFRs, identified through the FFN Hip Fracture Audit SIG, were consulted to assist in the identification of relevant HFRs from their areas of knowledge and expertise. Once an eligible HFR was identified, the registry website was searched for data dictionaries, guidance relating to administration of delirium and cognitive assessment tools, and the registry’s most recent annual report. Where the documentation was not published or was not available on the registry website, the relevant registry Chairs were contacted. Non-English registry documents were translated and included.

### Study selection

All citations identified from the collective search strategy were imported to Covidence (www.covidence.org) for reference management and de-duplication. Titles and abstracts of the remaining citations were independently reviewed by two reviewers, NAM and another author (ES, MEW) to identify those for full-text review. The full texts were obtained and independently evaluated by two reviewers, NAM and another author (RSP, MEW) applying the defined inclusion and exclusion criteria. Where disagreements occurred, discussions were held to reach consensus and where necessary, a third reviewer (CB) was involved. Citations excluded during the full-text review stage were documented alongside the reasoning for their exclusion and included in the PRISMA flow diagram [28].

### Data extraction

Data extraction was performed independently by two reviewers (NAM, RSP). Disagreements between reviewers were resolved through discussion or through consultation with a third reviewer (CB). Any missing details were sought through further contact with the relevant registry report authors. Table 2 outlines the data extracted from eligible registries.

**Table 2:** Hip fracture care items extracted from eligible registry documentation

*Hip fracture registry context and characteristics*
Setting
Age of patient inclusion
Years of registry operation
Year of latest published report and year of data reported
Types of cases included
Number of sites
Number of cases registered in latest report
Level of coverage (e.g., national, partial, large-scale)
Delirium standards of care or quality indicators (National Clinical Guidelines were out of scope)
Cognition standards of care or quality indicators (National Clinical Guidelines were out of scope)
Patient follow-up
Mandatory or voluntary participation
*Delirium and cognitive assessment data items*
Associated registry guidance for delirium and cognitive assessment tool administration
Question/specific tool
Response options (e.g., Yes/No/Unsure, score out of 12)
Timing of assessment in patient journey
Frequency of assessment
Healthcare professional responsible for assessment
Number of patients assessed at each timepoint
Number of patients who screened positive at each timepoint

### Data synthesis

Results were synthesised using descriptive statistics and/or narrative summaries where appropriate. A comprehensive table was created in which all eligible HFRs were listed, and their characteristics were presented. The data items and associated guidance for delirium and cognitive assessment that were included in each HFR were presented in tables. The delirium and cognitive assessment outcomes that were reported in the most recent annual reports of the identified registries (i.e., completeness of delirium and/or cognitive assessment, percentage of patients with positive screen) were also presented. For comparability with Johansen et al. [14], included registries were allocated to one of four discrete groups based on their origin and duration since becoming established: (1) first-generation registries (the longest established programmes originating from Scandinavia: Denmark; Finland; Norway; and Sweden); (2) second-generation registries (with structure based on the first-generation experience: Australia and New Zealand; England, Wales, and Northern Ireland; Germany, Austria, and Switzerland; Ireland; Netherlands; Scotland; and Spain); (3) third-generation registries (that have been recently established, are in an introductory period, or have not yet reached nationwide coverage: Argentina; China; Greece; Japan; Mexico; Pakistan; Philippines; and Portugal); and (4) other registries (whose structure was independent of the first-generation experience: Italy; Mexico; United States—KPHFR; United States—ACS-NSQIP).

## Results

### Study identification

**Fig. 1:**
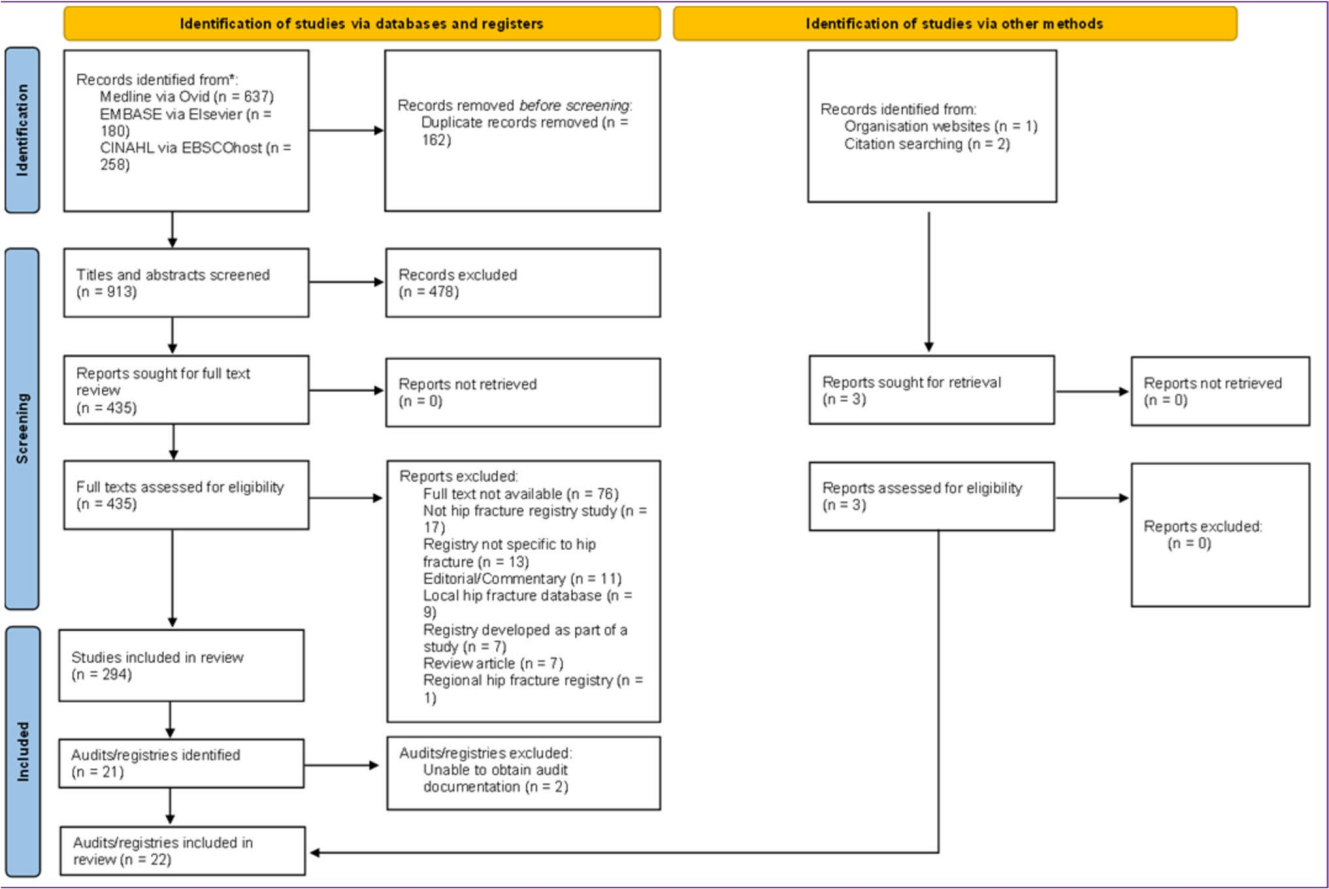
PRISMA flowchart diagram for the search and study selection process

The search of electronic databases identified a total of 1,075 citations. After de-duplication, 918 titles and abstracts were screened. A total of 435 full text records were assessed for eligibility in accordance with the inclusion and exclusion criteria, from which 22 different HFRs were identified. An additional three registries were identified from citation searches of previously published reviews and from the FFN website. Following further review, two registries were excluded as we were unable to obtain the relevant registry documentation and the registry leads did not respond (Lebanese Hip Fracture Registry [29] and Korean Hip Fracture Registry [30]) to information requests. The PRISMA flow diagram is presented in Fig. 1.

### Characteristics of registries

Twenty-two HFRs covering 27 countries were eligible for inclusion and their characteristics are presented in Table 3 (see Supplementary File S3 for summary of registry characteristics and Supplementary Table S4 for checklist of 125 most populated United Nations (UN) member states or constituent countries of a UN member state compared against identified HFRs). These registries include: American College of Surgeons National Surgical Quality Improvement Program Targeted Procedure Hip Fracture Dataset (ACS-NSQIP) (United States) [31];

**Table 3:** Characteristics of included hip fracture registries

Registry (Country)	Age of inclusion (years)	Year started	Year of latest published report (Year of data reported)	Patients registered	Hospital Sites	Fracture types	Level of coverage	Delirium Standards	Cognition Standards	Follow up (days)	Participation
ACS-NSQIP (United States)	18+	2016 for targeted procedure hip fracture dataset; 2004 for NSQIP (in current form)	N/R (2023)	11,570	111	1; 2; 3; 4; 5; Surgical management only; Pathological fractures excluded	Non-national: Voluntary participating hospitals only	N/R	N/R	30	Voluntary
AHFR (Argentina)	60+	2024	N/A	N/A	3	1; 2; 3; 4; 5; Pathological fractures excluded; High energy fractures excluded	National—under development	N/R	N/R	30; 120; 365	Voluntary
ANZHFR (Australia and New Zealand)	50+	2016	2024 (2023)	17,734 (All ANZHFR) 3,668 (New Zealand) 14,066 (Australia)	21 (New Zealand) 79 (Australia)	1; 2; 3; 4	National (88% eligible hospitals)	Yes	Yes	120	Voluntary
ATR-DGU (Germany, Austria, Switzerland)	70+	2016	2024 (2023)	16,361	145 (Germany) 2 (Austria) 6 (Switzerland)	1; 2; 3; 4; 5; Periprosthetic fractures included; Surgical management only	Not national, though participation is mandatory for all DGU geriatric trauma centres across Germany, Austria, and Switzerland	N/R	N/R	120	Mandatory
CHFR (China)	65+	2022	N/A	N/A	N/A	1; 2; 3; 4; 5; High energy fractures included; Pathological fractures excluded; Periprosthetic fractures excluded	National—under development	N/R	N/R	30; 120	Voluntary
DHFA (Netherlands)	18+	2016	2023 (2023)	18,918 (in report) 19,054 (total cases, including those registered after reporting cut-off)	66	1; 2; 3, 4; Pathological fractures excluded; Periprosthetic fractures excluded	National (66/71 hospitals—93% coverage) 97% of all fractures are recorded	Yes	N/R	90; 365	Voluntary
DMHFR (Denmark)	65+	2003	2023 (2023)	7,543 (7,539 included in analysis)	20	1; 2; 3; 4; Surgical management only	National (all trauma-receiving hospitals)	N/R	Yes	30; 365	Mandatory
Finland PERFECT (Finland)	50+	1999	N/A (2023)	7,041 (new hip fracture cases)	All hospitals in Finland from all 22 welfare counties	1; 2; 3; 4; Surgical management only	National (100% of cases treated in trauma-receiving hospitals are captured)	N/R	N/R	365	Mandatory
GFHFR (Greece)	60+	2022	2024 (Sept 2022—Dec 2023)	1,009	6	1; 2; 3; 4; 5; Pathological fractures excluded; High energy fractures excluded	National – introductory period	N/R	N/R	30	Voluntary
GIOG (Italy)	65+	2016	N/A (Jan 2020—Dec 2023)	2,842 (678 in 2023)	13	1; 2; 3; 4; 5; Surgical management only	Partial—GIOG participating centres	Yes	Yes	365	Voluntary
HipFRoP (Pakistan)	60+	2023	N/A	N/A	N/A	1; 2; 3; 4; 5	National—under development	N/R	N/R	30	Voluntary
IHFD (Ireland)	60+	2012	2023 (2023)	3,983 (in report) 4,025 (total cases, including those registered after reporting cut-off)	16	1; 2; 3; 4; 5; Pathological fractures excluded; High energy fractures excluded	National (all trauma-receiving hospitals)	N/R	N/R	30	Voluntary
IMSS (Mexico)	65+	2022	2024 (Nov 2022—Oct 2023)	1,042	24	1; 2; 3; 4; 5	National – introductory period	N/R	N/R	30; 120	Voluntary
JNHFD (Japan)	50+	2017	2024 (2017 – 2023)	46,019	> 400	1; 2; 3; 4; 5; Pathological fractures excluded; High energy fractures excluded	National—introductory period	N/R	N/R	30	Voluntary
KPHFR (United States)	0+	2009	2024 (2023)	6,803	36	1; 2; 3; 4; Surgical management only	Integrated healthcare system of Kaiser Permanente Federation hospitals covering > 11 million people throughout 8 US geographical Regions	N/R	N/R	Lifelong (Electronic Health Records)	Mandatory
NHFD (England, Wales, Northern Ireland)	60+	2007	2024 (2023)	72,466 (all NHFD) 65,843 (England) 4,336 (Wales) 2,287 (Northern Ireland)	169 (All NHFD) 153 (England) 12 (Wales) 4 (Northern Ireland)	1; 2; 3; 4; 5; Periprosthetic femoral fracture included; Fractures of greater or lesser trochanters excluded	National (all trauma-receiving hospitals)	Yes	N/R	30; 120	Voluntary
NHFR (Norway)	0+	2005	2024 (2023)	8,143 (primary operations) 807 (reoperations)	46	1; 2; 3; 4; Surgical management only	National (all trauma-receiving hospitals)	N/R	N/R	120; 365, 36 months	Voluntary
PHFRP (Philippines)	60+	2020	2023 (June 2020—Feb 2021)	158	12	1; 2; 3; 4; 5	National – introductory period	N/R	N/R	30	Voluntary
Rikshöft (Sweden)	50+	1988	2024 (2023)	10,800	38	1; 2; 3; 4	National (all trauma-receiving hospitals)	N/R	N/R	120	Voluntary
RNFA (Portugal)	65+	2025	N/A	N/A	10	1; 2; 3; 4; High energy fractures excluded	National—under development	N/R	N/R	30; 120	Voluntary
RNFC (Spain)	75+	2017	2025 (2023)	9,906 (9,467 who consented were included in analysis)	53	1; 2; 3; 4; High energy fractures excluded	National (53/449 hospitals in Spain, though not all of these are trauma-receiving)	Yes	N/R	30; 120	Voluntary
SHFA (Scotland)	50+	2013 (original 1993–2008)	2024 (2023)	8,355	17	1; 2; 3; 4; 5; Periprosthetic fractures excluded; Fractures of greater trochanters excluded	National (all trauma-receiving hospitals)	Yes	N/R	60	Voluntary

*Registries*: ACS-NSQIP—American College of Surgeons National Surgical Quality Improvement Program Targeted Procedure Hip Fracture Dataset; ATR-DGU—AltersTraumaRegister—Deutsche Gesellschaft für Unfallchirurgie; AHFR—Argentinian Hip Fracture Registry; ANZHFR—Australian and New Zealand Hip Fracture Registry; CHFR—Chinese Hip Fracture Registry; DMHFR—Danish Multidisciplinary Hip Fracture Registry; DHFA—Dutch Hip Fracture Audit; Finland PERFECT—Finland PERFormance, Effectiveness, and Costs of Treatment Hip Fracture Database; GFHFR—Greek Fragility Hip Fracture Registry; GIOG—Gruppo Italiano di Ortogeriatria; HipFRoP—Hip Fracture Registry of Pakistan; IHFD—Irish Hip Fracture Database; JNHFD—Japan National Hip Fracture Database; KPHFR—Kaiser Permanente Hip Fracture Registry; IMSS—Mexican Social Security Institute Multicentre Hip Fracture Registry; NHFD—National Hip Fracture Database; NHFR—Norwegian Hip Fracture Register; PHFRP—Philippine Hip Fracture Registry Project; RNFA—Portuguese National Registry of Hip Fractures; SHFA—Scottish Hip Fracture Audit; RNFC—Spanish Hip Fracture Registry; Rikshöft—Swedish National Hip Fracture Registry

*Fracture types*: 1—Intracapsular non-displaced; 2—Intracapsular displaced; 3—Trochanteric; 4—Subtrochanteric; 5—Other

*Additional abbreviations*: N/A—not applicable; N/R—not reported

**Fig. 2:**
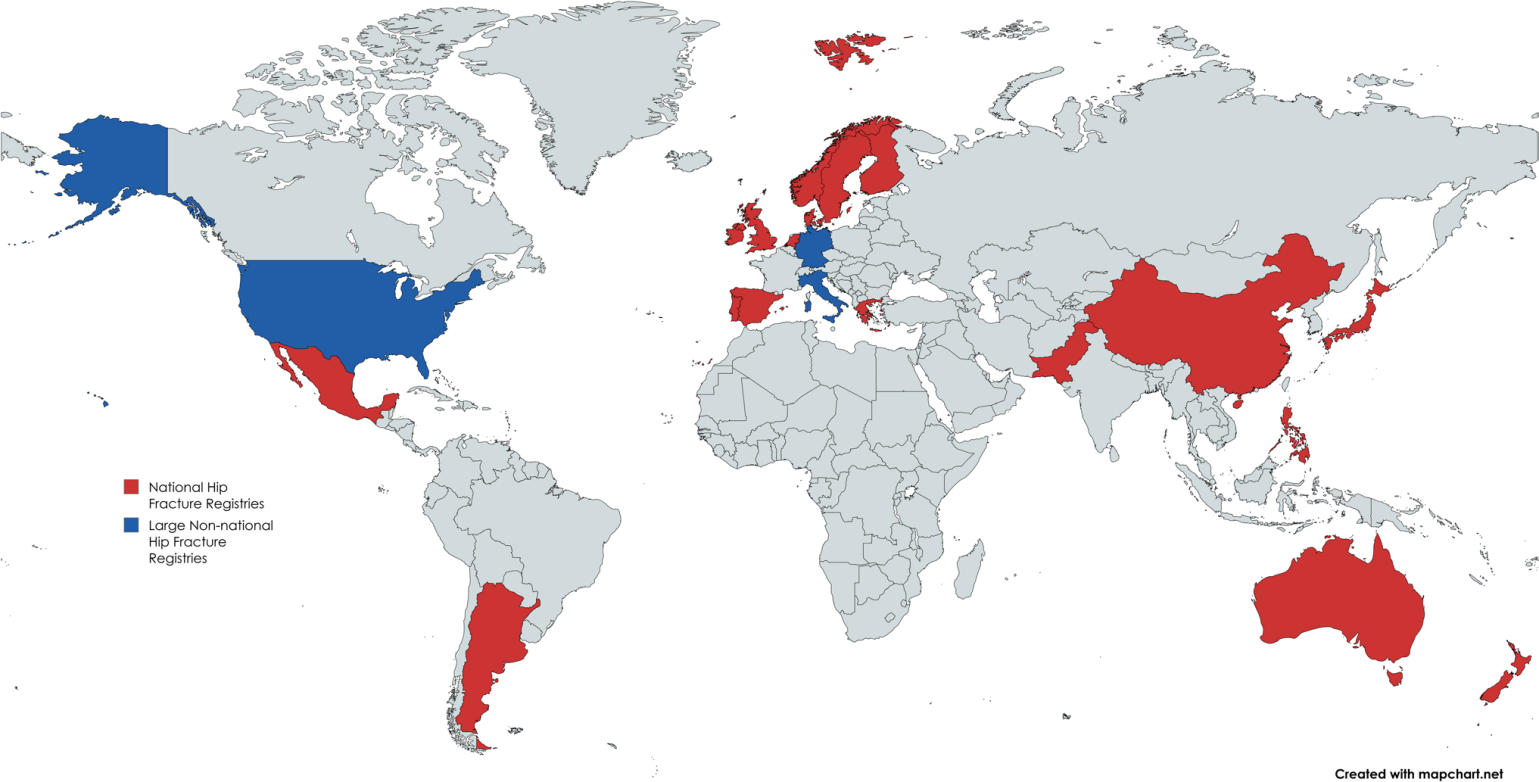
Graphical representation of the location of identified hip fracture registries

AltersTraumaRegister—Deutsche Gesellschaft für Unfallchirurgie (ATR-DGU) (Germany, Austria, Switzerland) [32]; Argentinian Hip Fracture Registry (AHFR) (Argentina) [33]; Australian and New Zealand Hip Fracture Registry (ANZHFR) (Australia, New Zealand) [34]; Chinese Hip Fracture Registry (CHFR) (China) [35]; Danish Multidisciplinary Hip Fracture Registry (DMHFR) (Denmark) [36]; Dutch Hip Fracture Audit (DHFA) (Netherlands) [37]; Finland PERFormance, Effectiveness, and Costs of Treatment Hip Fracture Database (Finland PERFECT) (Finland) [38]; Greek Fragility Hip Fracture Registry (GFHFR) (Greece) [39]; Gruppo Italiano di Ortogeriatria (GIOG) (Italy) [40]; Hip Fracture Registry of Pakistan (HipFRoP) (Pakistan) [41]; Irish Hip Fracture Database (IHFD) (Ireland) [27]; Japan National Hip Fracture Database (JNHFD) (Japan) [42]; Kaiser Permanente Hip Fracture Registry (KPHFR) (United States) [43]; Mexican Social Security Institute Multicentre Hip Fracture Registry (IMSS) (Mexico) [44]; National Hip Fracture Database (NHFD) (England, Wales, Northern Ireland) [45]; Norwegian Hip Fracture Register (NHFR) (Norway) [46]; Philippine Hip Fracture Registry Project (PHFRP) (Philippines) [47]; Portuguese National Registry of Hip Fractures (RNFA) (Portugal) [48]; Scottish Hip Fracture Audit (SHFA) (Scotland) [49]; Spanish Hip Fracture Registry (RNFC) (Spain) [50]; and Swedish National Hip Fracture Registry (Rikshöft) (Sweden) [51]. Figure 2 presents a world map highlighting the locations of each identified hip fracture registry.

### Delirium and cognition standards

Six registries reported hip fracture standards or quality indicators related to delirium. The relevant quality indicator of Australia and New Zealand referred to the proportion of patients with a hip fracture who have had an assessment of post-operative delirium in the week following surgery [16]. In England, Wales, and Northern Ireland, the key performance indicator included the percentage of patients who were assessed and found not to have delirium when screened promptly following surgery [52]. In Italy, the relevant performance indicator related to the daily assessment of pre-operative and post-operative delirium with the 4 ‘A’s Test (4AT) [53, 54]. In Spain, the percentage of patients with delirium assessment using the 4AT at some point during their hospital admission has been included in the registry as a key performance indicator since 2023 [19]. In Scotland, the quality standards referred to delirium assessment with the 4AT in the Emergency Department (ED) and within 24 h of admission [17]. In the Netherlands, recommended protocols from the structural quality indicators from the Dutch Healthcare Inspectorate were reported for delirium management in patients aged ≥ 70 [55]. Similarly, delirium risk was assessed on admission of hip fracture patients aged ≥ 70 in accordance with the Dutch National Safety Management System [55]. While no delirium care standard was reported by the registry in Denmark, the registry steering group reported working towards including the systematic use of a validated tool for delirium detection [36].

Three registries reported hip fracture standards or quality indicators related to cognitive assessment. In Australia and New Zealand, the quality indicator referred to the proportion of patients with a hip fracture who had their pre-operative cognitive status assessed [16]. In Denmark, the quality indicator included the proportion of patients who have been assessed for cognitive impairment occurring prior to the current fracture [56]. In Italy, the key performance indicator related to the assessment of pre-operative cognitive status.

### Delirium assessment data items

The description of delirium assessment practices across timepoints for the included registries is presented in Table 4.

**Table 4:** Description of delirium assessment practices across registries and reported percentages across time points

Registry name (country)	Delirium included	Pre-operative delirium assessed	Post-operative delirium assessed	Delirium measure	HCP responsible	Response options	Frequency of assessment	% Assessed pre-operatively/on admission	% Positive pre-operatively/on admission	% Assessed post-operatively	% Positive post-operatively
ACS-NSQIP (United States)	Yes	Yes (pre-operative)	Yes (post-operative)	Retrospective chart review	N/R	Yes; No; Missing	2				
AHFR (Argentina)	Yes	No	Yes (during first week following surgery)	CAM	MDT member	Yes; No; Unknown	Daily				
ANZHFR (Australia and New Zealand)	Yes	Yes (pre-operative)	Yes (during first week following surgery)	Validated Tool	N/R	Not assessed; Assessed and not identified; Assessed and identified; Not known	2			79.5	42.0
ATR-DGU (Germany, Austria, Switzerland)	Yes	Yes (on admission)	Yes (during hospital stay)	Nu-DESC	N/R	Yes; No; Unknown; Total, if yes	N/R				
CHFR (China)	Yes	No	Yes (post-operative)	N/R	N/R	N/R	N/R				
DHFA (Netherlands)	Yes	Peri-operative	Peri-operative	Registered as complication	MDT member	Yes; No; Missing	N/R			94.7	7.3
DMHFR (Denmark)	No										
Finland PERFECT (Finland)	No										
GFHFR (Greece)	No										
GIOG (Italy)	Yes	Yes (Day 0)	Yes (Day 1, 2, 3)	4AT	Geriatrician or orthopaedic surgeon	Out of 12; unknown	4	89.3 (Day 0)	18.2 (Day 0)	83.8 (Day 1) 74.4 (Day 2) 74.8 (Day 3)	22.7 (Day 1) 19.6 (Day 2) 18.2 (Day 3)
HipFRoP (Pakistan)	No										
IHFD (Ireland)	Yes	Yes (Day 1)	Yes (Day 3, Other)	4AT	Orthogeriatric team; Nursing staff; HSCP; NCHD	Yes; No; Unable; Undocumented If Yes, Scored out of 12	3	47.7 (Day 1)	14.7 (Day 1)	34.6 (Day 3) 38.6 (Other)	20.8 (Day 3) 22.3 (Other)
IMSS (Mexico)	Yes	Yes (pre-admission; pre-operative)	Yes (post-operative; daily)	CAM	Geriatrician and Nurse Specialist in Geriatrics	As per CAM	Daily	95.9 (pre-admission) 95.9 (pre-operative)	11.0 (pre-admission) 17.9 (pre-operative)	95.9	5.0
JNHFD (Japan)	No										
KPHFR (United States)	No										
NHFD (England, Wales, Northern Ireland)	Yes	Yes (pre-operatively on admission)	Yes (post-operative between 3rd and 7th day)	4AT	N/R	Out of 12; not done; patient refused	2				28.2
NHFR (Norway)	Yes	Peri-operative	Peri-operative	4AT	Nursing, Orthopaedic surgeons, Geriatricians	Yes; No; Missing	N/R				
PHFRP (Philippines)	No										
Rikshöft (Sweden)	No										
RNFA (Portugal)	Yes	Yes (on admission)	Yes (post-operative within 24 h)	4AT	MDT member	> = 4; 1–3; 0; Unknown	2				
RNFC (Spain)	Yes	Yes (on admission)	Yes (post-operative within 24 h)	4AT	N/R	> = 4; 1–3; 0; Unknown	2	68.0 (on admission)	22.0 (on admission)	72.7	23.8
SHFA (Scotland)	Yes	Yes (in ED; admission to admitting ward)	Yes (post-operative)	4AT	Medical or Nursing staff	Out of 12; unknown	3	66.7 (in ED) 72.7 (on ward admission)	17.5 (in ED) 21.5 (on ward admission)	34.2	32.0

*Registries*: ACS-NSQIP—American College of Surgeons National Surgical Quality Improvement Program Targeted Procedure Hip Fracture Dataset; ATR-DGU—AltersTraumaRegister—Deutsche Gesellschaft für Unfallchirurgie; AHFR—Argentinian Hip Fracture Registry; ANZHFR—Australian and New Zealand Hip Fracture Registry; CHFR—Chinese Hip Fracture Registry; DMHFR—Danish Multidisciplinary Hip Fracture Registry; DHFA—Dutch Hip Fracture Audit; Finland PERFECT—Finland PERFormance, Effectiveness, and Costs of Treatment Hip Fracture Database; GFHFR—Greek Fragility Hip Fracture Registry; GIOG—Gruppo Italiano di Ortogeriatria; HipFRoP—Hip Fracture Registry of Pakistan; IHFD—Irish Hip Fracture Database; JNHFD—Japan National Hip Fracture Database; KPHFR—Kaiser Permanente Hip Fracture Registry; IMSS—Mexican Social Security Institute Multicentre Hip Fracture Registry; NHFD—National Hip Fracture Database; NHFR—Norwegian Hip Fracture Register; PHFRP—Philippine Hip Fracture Registry Project; RNFA—Portuguese National Registry of Hip Fractures; SHFA—Scottish Hip Fracture Audit; RNFC—Spanish Hip Fracture Registry; Rikshöft—Swedish National Hip Fracture Registry

*Additional abbreviations*: 4AT—4 ‘A’s Test; CAM—Confusion Assessment Method; ED—Emergency Department; HCP—healthcare professional; HSCP—Health and Social Care Professions; MDT—multidisciplinary team; NCHD—Non-Consultant Hospital Doctor; N/R—not reported; Nu-DESC—Nursing Delirium Screening Scale

#### Pre- and post-operative delirium assessment

Fourteen out of 22 registries (63.6%) included a measure of delirium. Of these, one was first-generation (Norway), seven were second-generation (Australia and New Zealand; England, Wales, and Northern Ireland; Germany, Austria, and Switzerland; Ireland; Netherlands; Spain; and Scotland), three were third-generation (Argentina; China; and Portugal), and three were other registries (Italy; Mexico; and United States—ACS-NSQIP). Ten of the 14 registries (71.4%; 45.5% of all eligible registries) included a pre-operative measure of delirium: six second-generation registries (Australia and New Zealand; England, Wales, and Northern Ireland; Germany, Austria, and Switzerland; Ireland; Spain; and Scotland), one third-generation (Portugal), and three other registries (Italy; Mexico; and United States—ACS-NSQIP). Two registries (Australia and New Zealand; and England, Wales, and Northern Ireland) introduced pre-operative delirium assessments from 2024.

The reported timing of pre-operative delirium assessment varied across HFRs. Two registries assessed delirium at two timepoints pre-operatively. The Scottish registry assessed delirium at attendance to the ED and on admission to the admitting ward. The Mexican registry recorded delirium pre-admission and pre-operatively. Timing of pre-operative delirium assessment was recorded as either ‘on admission’ or ‘pre-operative’ across all 10 registries. However, one registry (Italy) specified the delirium assessment on admission as ‘Day 0’, while another registry (Ireland) specified the delirium assessment on admission as ‘Day 1’.

Twelve of the 14 registries (85.7%; 54.5% of all eligible registries) assessed delirium post-operatively: six were second-generation (Australia and New Zealand; England, Wales, and Northern Ireland; Germany, Austria, and Switzerland; Ireland; Scotland; and Spain), three were third-generation (Argentina; China; and Portugal), and three were other registries (Italy; Mexico; and United States—ACS-NSQIP). One registry (Norway) included a peri-operative delirium assessment from late 2023. One registry (Netherlands) recorded delirium as a complication during the hospital admission. One registry (Scotland) introduced a post-operative assessment of delirium in 2023.

The reported timing of post-operative delirium assessment also varied across HFRs. Four registries (China; Mexico; Scotland; and United States—ACS-NSQIP) did not specify a timeframe other than post-operatively. Two registries (Portugal; and Spain) specified a timeframe of 24 h post-operatively. Two registries (Argentina; and Australia and New Zealand) specified that post-operative delirium assessments should be conducted during the first week following surgery. One registry (Germany, Austria, and Switzerland) specified that post-operative delirium assessment should occur during the hospital stay. One registry (England, Wales, and Northern Ireland) recommended that the post-operative delirium assessment should occur between the third and seventh day. One registry (Italy) recommended post-operative delirium assessments on ‘Day 1’, ‘Day 2’ and ‘Day 3’, with ‘Day 0’ indicating the day of admission. One registry (Ireland) recommended post-operative delirium assessments on the third day of admission and at any other time during admission.

#### Delirium assessment tools

Seven registries (50%; 31.8% of all eligible registries) out of the 14 registries that assessed delirium (England, Wales, and Northern Ireland; Ireland; Italy; Norway; Portugal; Spain; and Scotland) recommended the 4AT assessment tool [54]. Two registries (Argentina; and Mexico) recommended the Confusion Assessment Method (CAM) [57] and one registry (Germany, Austria, and Switzerland) recommended the Nursing Delirium Screening Scale (Nu-DESC) [58]. One registry (Australia and New Zealand) recommended use of a validated tool to detect delirium but did not specify the tool. One registry (United States—ACS-NSQIP) identified delirium through retrospective chart review. One registry (Netherlands) recorded delirium as a medical complication, in addition to assessing delirium risk upon hospital admission of hip fracture patients aged ≥ 70. One registry (China) did not report the method of delirium assessment. Eleven registries included an option in the delirium assessment data field for unknown, missing, or not documented. Patient-related factors, such as ‘unable’ or ‘patient refused’ were also included as response options.

#### Healthcare professional responsible for delirium assessment

Four registries (Argentina; Ireland; Netherlands; and Portugal) reported that any member of the multidisciplinary team (MDT) could conduct delirium assessments. One registry (Scotland) reported that medical and nursing staff generally conduct delirium assessments. One registry (Mexico) specified that geriatricians and nurse specialists in geriatrics conducted delirium assessments. One registry (Italy) reported that geriatricians or orthopaedic surgeons conducted delirium assessments. One registry (Norway) reported that while orthopaedic surgeons input data to the registry, delirium assessment was conducted by nursing staff, orthopaedic surgeons, or geriatricians. The remaining registries did not specify which healthcare professionals were responsible for delirium assessments.

#### Prevalence of pre-operative delirium or delirium on admission

**Fig. 3:**
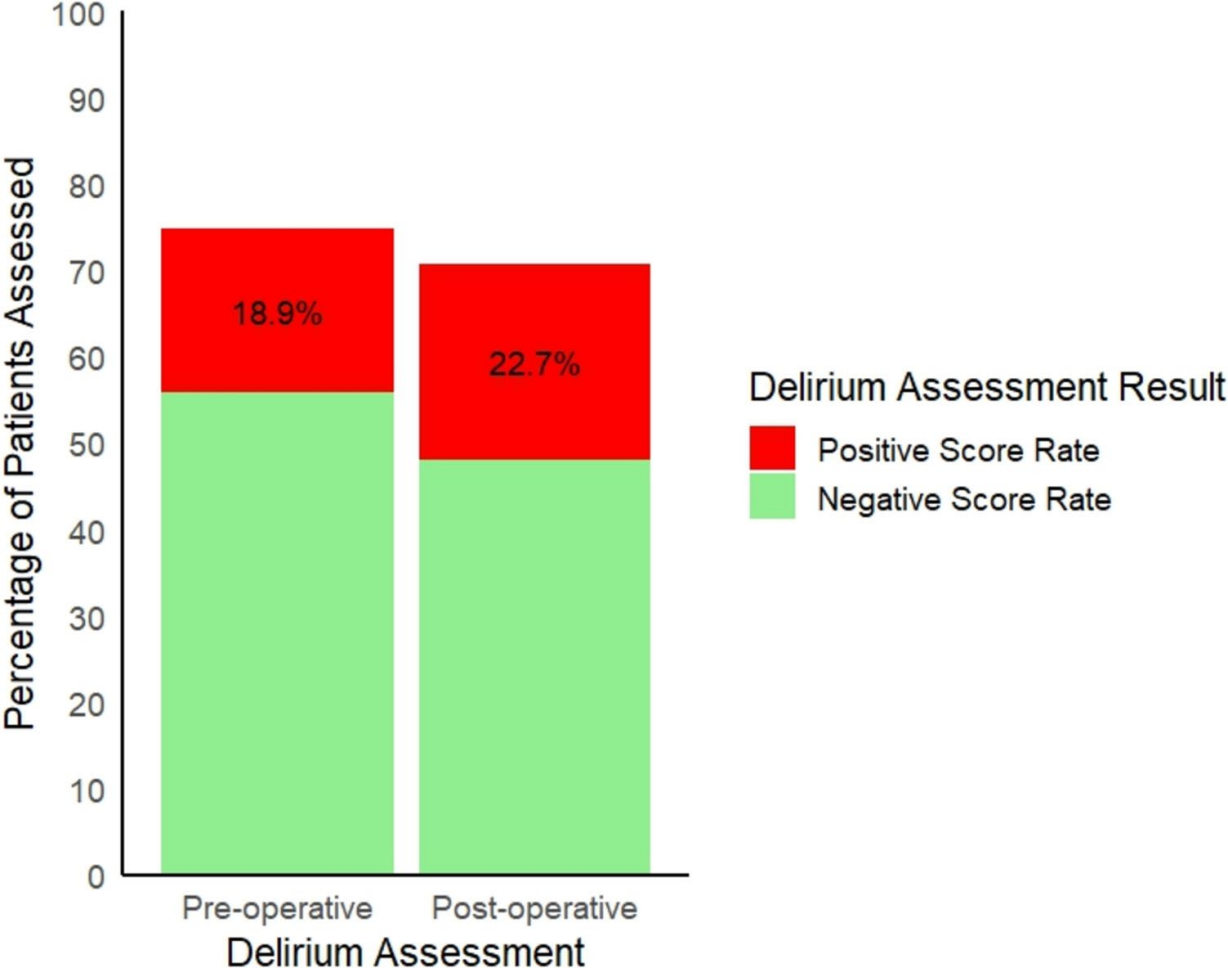
Mean delirium prevalence at pre- and post-operative assessment

Five registries (Ireland; Italy; Mexico; Scotland; and Spain) reported pre-operative (or on admission) tool completion rates. Two registries (Mexico; and Scotland) also reported tool completion rates for pre-admission delirium (95.9%) and delirium in the ED (66.7%), respectively. The mean reported pre-operative (or on admission) tool completion rate was 74.7% (47.7–95.9%). The mean percentage reported pre-operative (or on admission) positive score rate (delirium present) across those five registries was 18.9% (14.7–22%) (see Fig. 3). Across the four registries using the 4AT, the mean pre-operative (or on admission) positive score rate was 19.1% (14.7–22%).

#### Prevalence of post-operative delirium

Seven registries (Australia and New Zealand; Ireland; Italy; Mexico; Netherlands; Scotland; and Spain) reported post-operative tool completion rates. One registry (England, Wales and Northern Ireland) reported the post-operative tool completion rate though not the positive score rate. Two registries (Ireland; and Italy) also reported post-operative tool completion rates across additional timepoints. The mean post-operative tool completion rate was 70.8% (34.2–95.9%). The mean post-operative positive score rate across eight registries was 22.7% (5–42%) (see Fig. 3). Across the five registries using the 4AT, mean post-operative positive score rate was 25.5% (20.8–32%).

### Cognition data items

The description of cognitive assessment practices for the included registries is presented in Table 5.

**Table 5:** Description of cognition assessment practices and reported percentages across registries

Registry name (country)	Cognition included	Cognition measure	Thresholds for cognitive assessment tools	Response options	Pre-operatively/on admission	Post-operatively/discharge	HCP responsible	Frequency of assessment	% Assessed pre-operatively/on admission	% Positive
ACS-NSQIP (United States)	Yes	History of dementia (Retrospective chart review)		Yes; No	Yes	No	N/R	1		
AHFR (Argentina)	Yes	SPMSQ	N/R	Yes; No; Unknown	Yes	Yes	MDT member	2		
ANZHFR (Australia and New Zealand)	Yes	History of dementia; Validated tool		History of dementia: Normal cognition; Impaired cognition or known dementia; Not known Validated tool: not assessed; Assessed and normal; Assessed and abnormal or impaired; Not known	Yes	No	N/R	1	74.5	42.0 (positive on assessment tool) 37.0 (history of dementia)
ATR-DGU (Germany, Austria, Switzerland)	No									
CHFR (China)	Yes	N/R		N/R	Yes	No	N/R	1		
DHFA (Netherlands)	Yes	VMS memory problem question; History of dementia		Yes; No; Missing	Yes	No	N/R	1	77.2 (VMS) 83.2 (history of dementia)	29.9 (VMS) 14.4 (history of dementia)
DMHFR (Denmark)	Yes	Validated tool		Cognitively impaired; Not cognitively impaired; Unknown	Yes	No	Nursing staff or Geriatrician	1	79.9	
Finland PERFECT (Finland)	Yes	History of dementia		Yes; No	Yes	No	Neurologist (Primarily)	1	100.0	29.5
GFHFR (Greece)	Yes	History of dementia; Validated tool		Normal; Known dementia; Not known dementia but positive screen for cognitive impairment	Yes	No	N/R	1	98.1	9.9 (positive on assessment tool) 26.1 (history of dementia)
GIOG (Italy)	Yes	SPMSQ; AD-8	≥ 3 errors	SPMSQ: Out of 10; Unknown	Yes	No	Geriatrician or Orthopaedic surgeon	1	72.6	49.8
HipFRoP (Pakistan)	Yes	AMTS	N/R	Total score out of 10; Unknown	N/R	No	N/R	N/R		
IHFD (Ireland)	No									
IMSS (Mexico)	Yes	History of dementia; MiniCog	< 3	History of dementia: Yes; No MiniCog: Normal; Abnormal; Not valuable	Yes	No	Geriatric physician	1	95.9	34.3 (positive on assessment tool) 9.8 (history of dementia)
JNHFD (Japan)	Yes	AMTS	< 7	Total score out of 10; Not done/Patient refused; Not assessed	Yes	No	N/R	1	58.5	52.4
KPHFR (United States)	No									
NHFD (England, Wales, Northern Ireland)	Yes^a^	AMTS	< 7	Out of 10; Not done/ Patient refused	Yes	No	N/R	1	94.5	
NHFR (Norway)	Yes	History of dementia		Cognitive impairment: Yes; No; Uncertain	Yes	No	Orthopaedic surgeon or MDT member	N/R	98.2	27.7
PHFRP (Philippines)	Yes	AMTS	< 8	Out of 10; Not done/Patient refused	N/R	No	N/R	N/R	75.9	25.0
Rikshöft (Sweden)	Yes	SPMSQ	> 3 errors	Number of errors out of 10; All clear; Not fully orientated; Known dementia (must have dementia diagnosis for this response); Missing	Yes	No	Nursing staff	1	88.2	17.9 (positive on assessment tool) 15.2 (history of dementia)
RNFA (Portugal)	Yes	SPMSQ	> 3 errors	Number of errors out of 10; Not Performed/Patient Refused	Yes	No	Orthogeriatrician	1—2		
RNFC (Spain)	Yes	SPMSQ	> 3 errors	Number of errors out of 10; Not Performed/Patient Refused	Yes	No	MDT member	1—2	74.6	44.3
SHFA (Scotland)	No									

*Registries*: ACS-NSQIP—American College of Surgeons National Surgical Quality Improvement Program Targeted Procedure Hip Fracture Dataset; ATR-DGU—AltersTraumaRegister—Deutsche Gesellschaft für Unfallchirurgie; AHFR—Argentinian Hip Fracture Registry; ANZHFR—Australian and New Zealand Hip Fracture Registry; CHFR—Chinese Hip Fracture Registry; DMHFR—Danish Multidisciplinary Hip Fracture Registry; DHFA—Dutch Hip Fracture Audit; Finland PERFECT—Finland PERFormance, Effectiveness, and Costs of Treatment Hip Fracture Database; GFHFR—Greek Fragility Hip Fracture Registry; GIOG—Gruppo Italiano di Ortogeriatria; HipFRoP—Hip Fracture Registry of Pakistan; IHFD—Irish Hip Fracture Database; JNHFD—Japan National Hip Fracture Database; KPHFR—Kaiser Permanente Hip Fracture Registry; IMSS—Mexican Social Security Institute Multicentre Hip Fracture Registry; NHFD—National Hip Fracture Database; NHFR—Norwegian Hip Fracture Register; PHFRP—Philippine Hip Fracture Registry Project; RNFA—Portuguese National Registry of Hip Fractures; SHFA—Scottish Hip Fracture Audit; RNFC—Spanish Hip Fracture Registry; Rikshöft—Swedish National Hip Fracture Registry

*Additional abbreviations*: AD-8—Ascertain Dementia 8; AMTS—Abbreviated Mental Test Score; HCP—healthcare professional; MDT—multidisciplinary team; N/R—not reported; SPMSQ—Short Portable Mental Status Questionnaire; VMS—Veiligheidsmanagementsysteem [Safety Management System]

^a^The NHFD collects the results of all four domains of the 4AT separately. It stopped collecting the 10-item AMTS in 2024 and instead uses the 4-item ‘AMT4’ sub-domain of the 4AT as an indicator of cognitive impairment

#### Cognitive assessment

Of the 22 registries, 18 (82%) included a measure of cognitive function. Of these, four were first-generation (Denmark; Finland; Norway; and Sweden), four were second-generation (Australia and New Zealand; England, Wales, and Northern Ireland; Netherlands; and Spain), seven were third-generation (Argentina; China; Greece; Japan; Pakistan; Philippines; and Portugal), and three were other registries (Italy; Mexico; and United States—ACS-NSQIP). Pre-operative cognition function was recorded for the majority of registries (two registries from Pakistan and Philippines did not report the timing of the cognitive assessment), while one registry (Argentina) recorded an additional measure of cognitive function at discharge.

#### Cognitive assessment tools

Five registries (27.8%; 22.7% of all eligible registries) out of the 18 registries that assessed cognitive function (Argentina; Italy; Portugal; Spain; and Sweden) recommended using the Short Portable Mental Status Questionnaire (SPMSQ) [57]. Italy also recommended the Ascertain Dementia 8 (AD-8) assessment [59]. Four registries (22.2%; 18.2% of all eligible registries; England, Wales, and Northern Ireland; Japan; Pakistan; and Philippines) recommended assessing cognitive function with the Abbreviated Mental Test Score (AMTS) assessment tool [60]. Four registries (Finland; Netherlands; Norway; and United States—ACS-NSQIP) reported history of dementia. Two registries (Australia and New Zealand; and Greece) reported history of dementia in addition to recommending the use of a validated tool. One registry (Mexico) reported history of dementia and recommended the use of the Mini-Cog assessment [61]. One registry (Denmark) recommended the use of a validated tool, though did not specify which tool. One registry (China) did not specify how cognition was assessed. One registry (Germany, Austria, and Switzerland) included the Identification of Seniors at Risk (ISAR) assessment tool [62], through which the presence of serious memory problems can be ascertained. As part of the Dutch National Safety Management System [55] in assessing delirium risk, the Netherlands registry also included the presence of memory problems pre-operatively in patients aged ≥ 70. From 2024, the registry from England, Wales, and Northern Ireland replaced the recording of pre-operative AMTS with the 4AT. Thirteen registries included an option in the cognitive assessment data field for unknown, missing, or not documented responses. Patient-related factors, such as ‘unable’ or ‘patient refused’ were also included as response options.

In terms of thresholds used for the specified cognitive assessment tools, three registries (Portugal; Spain; and Sweden) defined cognitive impairment as > 3 errors on the SPMSQ, while one registry (Italy) defined impaired cognition as ≥ 3 errors. One registry (Argentina) was in the process of establishing formal assessment thresholds. Two registries (England, Wales, and Northern Ireland; and Japan) defined impaired cognition as a score of < 7 on the AMTS, while one registry (Philippines) defined impaired cognition as < 8. One registry (Pakistan) did not report the threshold used for the AMTS. One registry (Mexico) defined cognitive impairment as a score of < 3 on the Mini-Cog.

#### Healthcare professional responsible for cognitive assessment

Two registries (Argentina; and Spain) reported that any member of the MDT could conduct a cognitive assessment. One registry (Norway) reported that orthopaedic surgeons or the MDT were responsible for conducting cognitive assessments. One registry (Mexico) reported that geriatricians were responsible for cognitive assessments. One registry (Portugal) reported that orthogeriatricians were responsible for conducting cognitive assessments. One registry (Italy) reported that geriatricians or orthopaedic surgeons conducted cognitive assessments. One registry (Denmark) reported that geriatricians or nursing staff conducted cognitive assessments. One registry (Sweden) reported that nursing staff were responsible for conducting cognitive assessments. One registry (Finland) reported that neurologists were primarily responsible for assessing cognitive function in the form of dementia diagnosis.

#### Prevalence of cognitive impairment or dementia

**Fig. 4:**
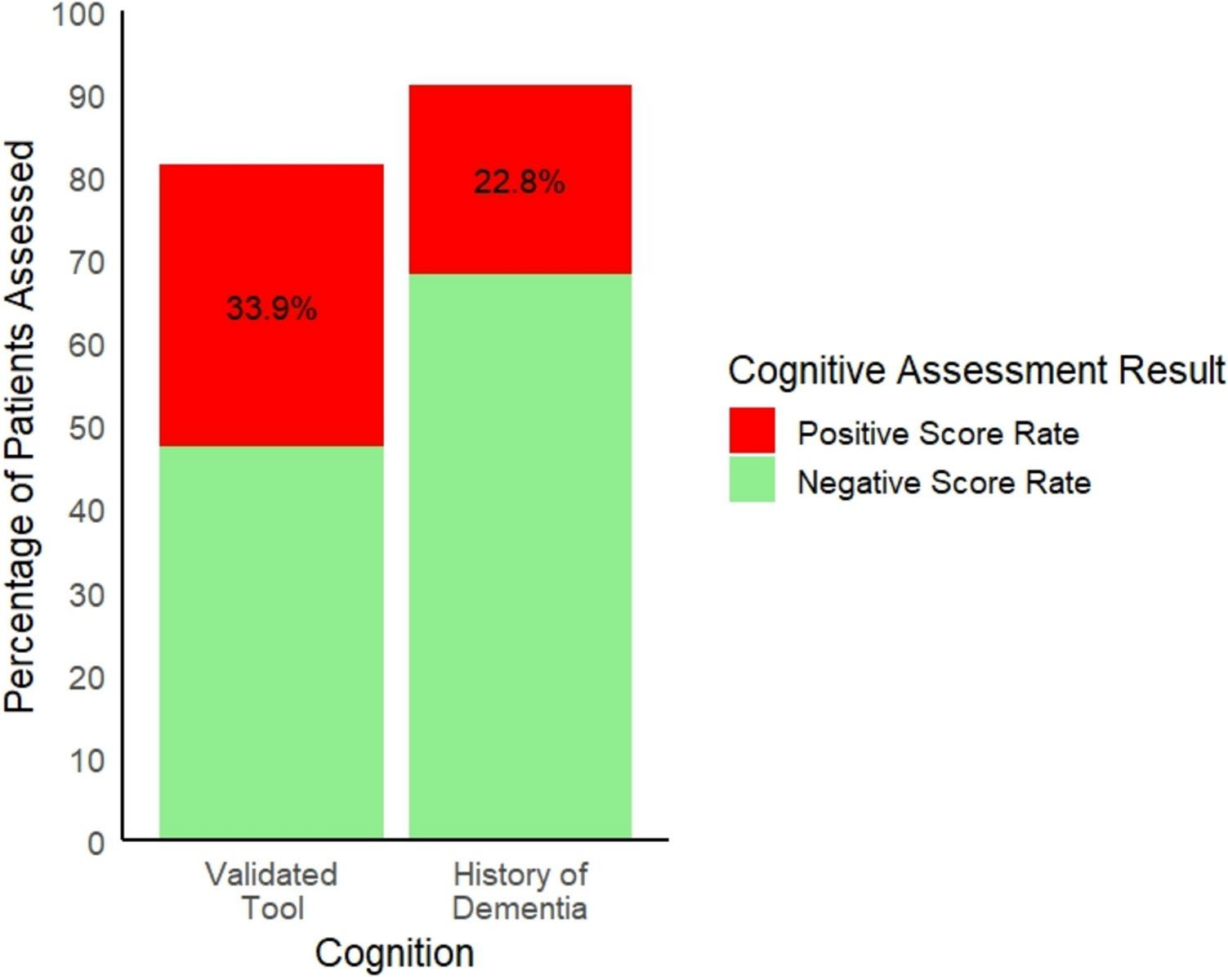
Mean prevalence of cognitive impairment or dementia across national hip fracture registries

Thirteen registries (Australia and New Zealand; England, Wales, and Northern Ireland; Denmark; Finland; Greece; Italy; Japan; Mexico; Netherlands; Norway; Philippines; Spain; and Sweden) reported cognitive assessment tool completion rates. The mean tool completion rate (including history of dementia) was 84.2% (58.5–100%). In the Netherlands registry, memory problems were ascertained in 77.2% of older adults aged ≥ 70 as part of the Safety Management System assessment, and history of dementia on admission was established for 83.2% of all adults aged ≥ 18. The overall mean positive score rate across 11 registries was 31.6% (9.9–52.4%). The mean positive score rate for validated cognitive assessment tools across nine registries was 33.9% (9.9% to 52.4%). The mean percentage of patients with a history of dementia across seven registries was 22.8% (9.8–37%) (see Fig. 4). Across two registries reporting data using the SPMSQ (with a cut off of > 3 errors), the mean positive score rate was 31.1% (17.9–44.3%).

## Discussion

This scoping review identified 22 national or large-scale non-national HFRs across 27 countries. Of these, 14 (64%) collected delirium assessment data, 18 (82%) collected cognitive assessment data, while only one registry collected neither. The 4AT was the most widely used delirium assessment across HFRs (50%). While 100% of first-generation, third-generation, 75% of other registries, and 57% of second-generation registries collected cognitive assessment data, 25% of first-generation, 100% of second-generation, 43% of third-generation, and 75% of other registries collected delirium assessment data. Hence, most HFRs are incorporating delirium and cognitive assessment data items, but there is heterogeneity in tools and methods. This variability spans several key areas, including the specific tools used for detection, the timing and frequency of assessments, the cut-off scores applied, tool completion rates, positive score rates, and the healthcare professionals responsible for assessment.

The MCD recommended by the FFN includes cognitive status assessment as a core component. However, no specific recommendations were made regarding assessment tools, timing, or frequency due to challenges such as language barriers, lack of locally validated tools, and cultural variations between countries [13]. When comparing data across different registries, variations in definitions and assessment methods can create difficulties. Therefore, it was recommended that registries provide detailed data dictionaries to ensure clear interpretation of their data [13]. These challenges were reflected in the current findings. While a substantial proportion of registries (50%) recommended the 4AT for delirium assessment, other tools such as the CAM and Nu-DESC were also recommended. These three tools were previously reported to have a pooled sensitivity of 0.76, 0.78, and 0.80, respectively and specificity of 0.90, 0.90, and 0.99, respectively for detecting delirium in hospitalised older adults [63]. However, post-operative delirium prevalence as measured by the CAM in the one registry (Mexico) reporting CAM figures was only 5%, much lower than expected levels. This may reflect the challenges in managing delirium in emerging economies, where surgery may be delayed, highlighting the importance of considering the time since fracture when assessing delirium. Furthermore, appropriate implementation of the CAM requires rigorous training, which limits its feasibility for routine use in HFRs [64–66]. The known low sensitivity of the CAM when used in routine practice [67] can be due to its use by inexperienced and minimally trained raters [64].

Similarly, cognitive function was assessed using a range of tools, with the SPMSQ and AMTS being the most common, as well as recording of a clinical diagnosis of dementia. Additionally, varying response options and scoring systems across registries made direct comparisons challenging. For example, when using the AMTS, registries defined impaired cognition as a score of less than 7 or less than 8. The healthcare professionals responsible for administering these assessment tools also varied across registries, ranging from any member of the MDT to more specialised professionals, such as geriatricians, nurse specialists in geriatrics, orthopaedic surgeons, or neurologists, depending on the assessment method. Adding to this complexity, the timing of pre- and post-operative delirium assessment varied considerably. For example, some registries specified assessment on presentation to the Emergency Department, on hospital admission, on ‘Day 0’ or on ‘Day 1’ (which both effectively meant pre-operatively), or simply ‘pre-operatively’. Employing a common definition and data field for pre-operative delirium assessment across registries would greatly enhance international comparability.

This heterogeneity poses challenges for comparing data and benchmarking performance across registries. The age of patient inclusion in HFRs is likely to impact on reported positivity rates for delirium and cognitive assessments. In addition, different tools have varying diagnostic accuracy, and the timing of assessment, cut-off scores, as well as potential biases in completion rates can significantly impact positive score rates [67]. This is evident in the wide ranges observed for both delirium and cognitive impairment positive score rates. For example, pre-operative delirium positive score rates ranged from 14.7 to 22% while postoperative rates varied more substantially, with ranges from 5 to 42%, though registries using the 4AT showed more consistent results (20.8–32%). One registry (England, Wales, Northern Ireland) currently recommends the 4AT for both delirium and cognitive assessment. While the 4AT is primarily designed as a rapid assessment tool for delirium rather than a comprehensive cognitive assessment [68], it includes the Abbreviated Mental Test 4 (AMT4) [69] and attention-related items that evaluate cognitive impairment, making it useful for detecting underlying dementia [70]. The cognitive components of the 4AT (AMT4 and attention items) exhibit high specificity but low sensitivity, as well as a high negative predictive value but low positive predictive value for diagnosing dementia [70, 71]. This suggests that a 4AT score of 1–3, based on cognitive test items and taking into account the availability of collateral history, should prompt concern about potential cognitive impairment but does not rule it out [71]. Therefore, if 4AT scores are collected rather than a dichotomous response of delirium present or absent, then it is possible for this tool to be used for both delirium and cognitive assessment.

Delirium remains under-detected in routine care, highlighting a major unmet clinical need [67]. This was also evident in the current scoping review. Tool completion rates exhibited substantial variability, ranging from 47.7 to 95.9% for pre-operative delirium assessment and 34.2–95.9% for post-operative delirium assessment. As suggested by a number of qualitative studies, this is likely influenced by factors such as time constraints, availability of collateral information, staff awareness and training, tool complexity, the point in the patient journey when the tool is administered, and cultural factors [67, 72–74]. Lower completion rates could indicate difficulties in tool administration by clinical staff or the tool not being attempted for various reasons. The variability and lower completion rates also may introduce a bias in the positive score rates, depending on the reasons for non-completion. A key challenge identified was the variation in how registries classify and record assessment results. The majority of registries include options for "unable to assess" or "unknown," which can potentially affect both completion and positive score rates [67].

This review also reveals encouraging progress in the collection of data related to cognitive impairment and delirium in international HFRs despite the challenges posed by heterogeneity. Compared to 65% in 2023 [14], a high percentage of registries now include a measure of pre-operative cognitive function (82%), demonstrating high levels of consistency among national programmes in this regard. Notably, 64% of registries now collect delirium assessment data, with additional registries contemplating the inclusion of delirium standards of care. Furthermore, the number of registries reporting delirium prevalence has increased from just two registries in a previous 2022 study examining differences across European HFRs to five registries in this review, suggesting a growing awareness and emphasis on delirium assessment [75]. This reflects a growing recognition of the importance of detecting pre-operative delirium or cognitive impairment in understanding patient risk, guiding management, and predicting outcomes. The increasing inclusion of delirium assessment as a standard of care is also a positive trend.

This scoping review benefited from a comprehensive search strategy, encompassing a wide range of databases and grey literature sources, which was supplemented through engagement with knowledge users. Furthermore, the inclusion of international registries enhances the generalisability of the findings and allows for a broader perspective on the challenges and opportunities in hip fracture registry data collection. However, the heterogeneity in the included registries made it challenging to synthesise the data and draw definitive conclusions. A limitation of our study is that we did not assess whether the tools used in each HFR were formally validated or appropriately translated for the local language and cultural context. Evaluating whether the chosen tools were validated for local use would be an important additional consideration for future studies.

For clinical practice, these findings underscore the importance of systematic cognitive and delirium assessment in hip fracture care while highlighting the need for more standardised approaches to improve cross-registry comparability. The emerging preference for certain tools (such as the 4AT) suggests potential for greater alignment in assessment practices. Current trends indicate that routine measurement of delirium and cognition both pre- and post-operatively is becoming increasingly important. It is of note that the NHFD has adopted the 4AT for both simple cognitive (scores 1–3) and delirium assessment (score 4+), streamlining assessment processes by requiring only one tool. Furthermore, the 4AT is already the most widely used tool in HFRs and is the most validated delirium assessment tool, supported by 33 studies involving over 6,000 patients—twice as many as the CAM or Nu-DESC [54]. Barriers to the implementation of the 4AT have been identified, including lack of awareness that the 4AT can be used with patients who are not alert, the need for tool validation in the local language, and lack of awareness that the 4AT can assist in differentiating delirium and pre-existing cognitive disorders, suggesting that development of educational materials to address these modifiable barriers may improve implementation rates [76]. Therefore, widespread adoption of the 4AT by registries could facilitate standardisation and enhance international comparability, ultimately improving the consistency and reliability of delirium and cognitive assessments in clinical practice.

## Conclusion

This scoping review demonstrated that the majority of identified HFRs included a measure of cognitive function, and nearly two thirds incorporated delirium assessment, reflecting increased awareness of the need to identify pre-operative delirium or cognitive impairment to assess patient risk, inform management strategies, and predict outcomes. Despite this progress, significant heterogeneity exists across registries in the tools used, scoring methods, completion rates, the healthcare professionals involved, and positive score rates. This variability hinders data comparison and benchmarking efforts. The growing preference for certain tools, such as the 4AT, suggests a potential pathway towards greater alignment in assessment practices. Future research and collaborative efforts should focus on standardising assessment protocols, improving tool completion rates, and addressing the challenges posed by varying data collection methods to enhance the comparability of data across HFRs, ultimately improving patient care.

## Supplementary Information

Below is the link to the electronic supplementary material.

Supplementary file1 (DOCX 41 KB)
