# Supplementary material for: Delirium and cognitive assessment in national hip fracture registries: a scoping review
Source: Eur Geriatr Med. 2025 Jun 6;16(5):1679–701. doi: 10.1007/s41999-025-01246-4 (PMC12528335; doi:10.1007/s41999-025-01246-4)
Supplement: Supplementary file 1 — Supplementary file1 (DOCX 41 KB) [file 41999_2025_1246_MOESM1_ESM.docx]

**Delirium and cognitive assessment in national hip fracture registries: A scoping review**

**European Geriatric Medicine**

**SUPPLEMENTARY INFORMATION**

**Author names:**

Niamh A. Merriman^1^

Rose S. Penfold^2^

Mary E. Walsh^1,3^

Eithne Sexton^4^

Louise Brent^4,5^

Pamela Hickey^5^

Tara Coughlan^6,7^

Cristina Ojeda-Thies^8,9^

Antony Johansen^10,11^

Andrew J. Hall^12^

Alasdair M. J. MacLullich^2^

Niamh O’Regan^13^

Catherine Blake^1^

On behalf of National Collaborators

**Corresponding author:**

Dr Niamh A. Merriman

School of Public Health, Physiotherapy and Sports Science,

University College Dublin,

Dublin,

Ireland

Email: [niamh.merriman1@ucd.ie](mailto:niamh.merriman1@ucd.ie)

**Supplementary Table S1.** Completed PRISMA-ScR checklist

**Supplementary Table S2.** Search Strategy

**Supplementary File S3**: Summary of characteristics of identified hip fracture registries

**Supplementary Table S4**: Checklist of 125 most populated United Nations (UN) member states or constituent countries of a UN member state against identified hip fracture registries

**Supplementary Table S1:** Preferred Reporting Items for Systematic reviews and Meta-Analyses extension for Scoping Reviews (PRISMA-ScR) Checklist

| **SECTION** | **ITEM** | **PRISMA-ScR CHECKLIST ITEM** | **REPORTED ON PAGE #** |
| --- | --- | --- | --- |
| **TITLE** | | | |
| Title | 1 | Identify the report as a scoping review. | 1 |
| **ABSTRACT** | | | |
| Structured summary | 2 | Provide a structured summary that includes (as applicable): background, objectives, eligibility criteria, sources of evidence, charting methods, results, and conclusions that relate to the review questions and objectives. | 5 |
| **INTRODUCTION** | | | |
| Rationale | 3 | Describe the rationale for the review in the context of what is already known. Explain why the review questions/objectives lend themselves to a scoping review approach. | 6 - 7 |
| Objectives | 4 | Provide an explicit statement of the questions and objectives being addressed with reference to their key elements (e.g., population or participants, concepts, and context) or other relevant key elements used to conceptualize the review questions and/or objectives. | 7 |
| **METHODS** | | | |
| Protocol and registration | 5 | Indicate whether a review protocol exists; state if and where it can be accessed (e.g., a Web address); and if available, provide registration information, including the registration number. | 7 |
| Eligibility criteria | 6 | Specify characteristics of the sources of evidence used as eligibility criteria (e.g., years considered, language, and publication status), and provide a rationale. | 8 |
| Information sources* | 7 | Describe all information sources in the search (e.g., databases with dates of coverage and contact with authors to identify additional sources), as well as the date the most recent search was executed. | 8 |
| Search | 8 | Present the full electronic search strategy for at least 1 database, including any limits used, such that it could be repeated. | 8 |
| Selection of sources of evidence† | 9 | State the process for selecting sources of evidence (i.e., screening and eligibility) included in the scoping review. | 9 |
| Data charting process‡ | 10 | Describe the methods of charting data from the included sources of evidence (e.g., calibrated forms or forms that have been tested by the team before their use, and whether data charting was done independently or in duplicate) and any processes for obtaining and confirming data from investigators. | 9 - 10 |
| Data items | 11 | List and define all variables for which data were sought and any assumptions and simplifications made. | 9 - 10 |
| Critical appraisal of individual sources of evidence§ | 12 | If done, provide a rationale for conducting a critical appraisal of included sources of evidence; describe the methods used and how this information was used in any data synthesis (if appropriate). | N/A |
| Synthesis of results | 13 | Describe the methods of handling and summarizing the data that were charted. | 10 - 11 |
| **RESULTS** | | | |
| Selection of sources of evidence | 14 | Give numbers of sources of evidence screened, assessed for eligibility, and included in the review, with reasons for exclusions at each stage, ideally using a flow diagram. | 11 |
| Characteristics of sources of evidence | 15 | For each source of evidence, present characteristics for which data were charted and provide the citations. | 12 |
| Critical appraisal within sources of evidence | 16 | If done, present data on critical appraisal of included sources of evidence (see item 12). | N/A |
| Results of individual sources of evidence | 17 | For each included source of evidence, present the relevant data that were charted that relate to the review questions and objectives. | 13 - 31 |
| Synthesis of results | 18 | Summarize and/or present the charting results as they relate to the review questions and objectives. | 13 - 31 |
| **DISCUSSION** | | | |
| Summary of evidence | 19 | Summarize the main results (including an overview of concepts, themes, and types of evidence available), link to the review questions and objectives, and consider the relevance to key groups. | 32 |
| Limitations | 20 | Discuss the limitations of the scoping review process. | 34 |
| Conclusions | 21 | Provide a general interpretation of the results with respect to the review questions and objectives, as well as potential implications and/or next steps. | 35 |
| **FUNDING** | | | |
| Funding | 22 | Describe sources of funding for the included sources of evidence, as well as sources of funding for the scoping review. Describe the role of the funders of the scoping review. | 2 |

**Supplementary Table S2**: Search Strategy

|  | **Medline Ovid** |
| --- | --- |
| 1 | Hip Fractures/ OR hip fracture*.ab,ti. |
| 2 | ((Hip fracture adj3 registry) OR (hip fracture adj3 registries) OR (National Hip Fracture Registry) OR (National Hip Fracture Register) OR (Hip fracture adj3 database) OR (Hip fracture adj3 audit*)).ab,ti. |
| 3 | ((quality register).ab,ti. OR (quality registry).ab,ti. OR (performance indicator*).ab,ti. OR (quality indicator*).ab,ti.) AND (hip fracture).ab,ti. |
| 4 | ((Audit adj2 guideline*) OR (registry adj2 guideline*)).ab,ti. |
| 5 | 2 OR 3 OR 4 |
| 6 | 1 AND 5 |

|  | **EMBASE on Elsevier.com** |
| --- | --- |
| 1 | ‘hip fracture$’:ti,ab OR 'hip fracture'/exp |
| 2 | ‘hip fracture’ NEXT/3 registry OR ‘hip fracture’ NEXT/3 registries OR ‘National Hip Fracture Registry’:ti,ab OR ‘National Hip Fracture Register’:ti,ab OR ‘hip fracture’ NEXT/3 audit$ |
| 3 | ((‘quality register’:ti,ab OR ‘quality registry’:ti,ab OR ‘performance indicator*’:ti,ab OR ‘quality indicator*’:ti,ab) AND ‘hip fracture’:ti,ab,de,kw) |
| 4 | (Audit NEXT/2 guideline$) OR (registry NEXT/2 guideline$) |
| 5 | 2 OR 3 OR 4 |
| 6 | 1 AND 5 |
| 7 | #6 **AND [humans]/lim AND [english]/lim** |
| 8 | **#7** AND [medline]/lim |
| 9 | #7 NOT #8 |

|  | **CINAHL** |
| --- | --- |
| 1 | (MH "Hip Fractures+") OR TI ‘hip fracture$’ OR AB ‘hip fracture$’ |
| 2 | (‘hip fracture’ N3 registry) OR (‘hip fracture’N3 registries) OR TX ‘National Hip Fracture Registry’ OR TX ‘National Hip Fracture Register’ OR (‘hip fracture’ N3 audit$) |
| 3 | (TX ‘quality register’ OR TX ‘quality registry’ OR TX ‘performance indicator$’ OR TX ‘quality indicator$’) AND (TI ‘hip fracture$’ OR AB ‘hip fracture$’) |
| 4 | (Audit N2 guideline$) OR (registry N2 guideline$) |
| 5 | S2 or S3 or S4 |
| 6 | S1 and S5 |
| 7 | Limit to English |

**Supplementary File S3**: Summary of characteristics of identified hip fracture registries

Published reports describing data from years 2017 to 2023 were available for 14 registries (United States–KPHFR; Netherlands; Denmark; Ireland; Philippines; Australia and New Zealand; Germany, Austria, and Switzerland; Greece; Mexico; Japan; England, Wales, and Northern Ireland; Norway; Sweden; and Scotland). Information regarding data dictionaries and/or data for the year 2023 was supplied via email correspondence by six registries (Argentina; Finland; Italy; Pakistan; Portugal; and Spain), for which no public report was available. Ten registries (United States–KPHFR; Netherlands; Denmark; Ireland; Germany, Austria, and Switzerland; Greece; Mexico; Japan; Norway; and Sweden) supplied supplemental information to published reports. Four registries (Argentina; China; Pakistan; and Portugal) were under development and as such did not have data to report. For one registry (United States–ACS-NSQIP), no annual report was available apart from the 2023 data dictionary.

The mean age since inception of the first-, second-, third-generation, and other registries was 26 years (20 to 37), 11 years (8 to 18), three years (0 to 8), and nine years (3 to 16) respectively. The oldest registry was from Sweden (37 years) and six of the most recent registries (Argentina; China; Greece; Mexico; Pakistan; and Portugal) had been established for three years or less.

Ten registries (Australia and New Zealand; Denmark; England, Wales, and Northern Ireland; Finland; Ireland; Netherlands; Norway; Scotland; Spain; and Sweden) reported a nationwide level of coverage. Four registries (Greece; Japan; Philippines; and Mexico) were in the introductory period and had not yet achieved national coverage. Four registries (Argentina; China; Pakistan; and Portugal) were under development. Four registries did not have national coverage: From the United States, ACS-NSQIP reported data from 994,313 cases submitted from 676 NSQIP-participating sites in 2023 and KPHFR reported coverage from the integrated healthcare system of Kaiser Permanente Federation hospitals covering >11 million people throughout eight US geographical regions. The ATR-DGU did not report national coverage, though participation is mandatory for all DGU geriatric trauma centres across Germany, Austria, and Switzerland. The registry in Italy had partial coverage through GIOG participating centres.

Two registries (Norway; and United States–KPHFR) included patients of any age and two registries (Netherlands; and United States–ACS-NSQIP) included patients aged 18 years and older. Five registries (Australia and New Zealand; Finland; Japan; Sweden; and Scotland) included patients aged 50 years and over, six registries (Argentina; Greece; Pakistan; Ireland; England, Wales, and Northern Ireland; and Philippines) included patients aged 60 and above, and five registries (China; Denmark; Italy; Mexico; and Portugal) included patients aged 65 and over. One registry (Germany, Austria, and Switzerland) included patients aged 70 and over, and one registry (Spain) included patients aged 75 and over.

Seven registries (United States–ACS-NSQIP; United States–KPHFR; Denmark; Germany, Austria, and Switzerland; Finland; Italy; and Norway) included surgically managed hip fractures only. Six registries (Argentina; Greece; Ireland; Japan; Portugal; and Spain) explicitly excluded high energy fractures, while one registry (China) included high energy fractures. Seven registries (Argentina; China; Greece; Ireland; Japan; Netherlands; and United States–ACS-NSQIP) explicitly excluded pathological fractures. Three registries (China; Netherlands; and Scotland) explicitly excluded peri-prosthetic fractures. Two registries (England, Wales, and Northern Ireland; and Scotland) excluded isolated greater and/or lesser trochanteric fractures.

Six registries (United States–ACS-NSQIP; Greece; Ireland; Japan; Pakistan; and Philippines) had follow-up of 30 days. Five registries (China; England, Wales, and Northern Ireland; Mexico; Portugal; and Spain) had follow-up periods of 30 and 120 days. Three registries (Australia and New Zealand; Germany, Austria, and Switzerland; and Sweden) had follow-up of 120 days. Two registries (Finland and Italy) had follow-up times of 365 days. One registry (Scotland) had follow-up of 60 days. One registry (Argentina) had follow-up periods of 30, 120, and 365 days. One registry (Denmark) had 30- and 365-day follow-up periods. The Netherlands registry had 90- and 365-day follow-up periods. One registry (Norway) had follow-up periods of 120 days, 365 days, and 36 months. One registry (United States–KPHFR) had lifelong follow-up.

The majority of the included registries had voluntary participation, while four registries (Denmark; Germany, Austria, and Switzerland; Finland; and United States–KPHFR) had mandatory participation.

**Supplementary Table S4**: Checklist of 125 most populated United Nations (UN) member states or constituent countries of a UN member state against identified hip fracture registries

| **Ranking** | **Country** | **Hip Fracture Registry Identified** |
| --- | --- | --- |
| 1 | India |  |
| 2 | China | Yes |
| 3 | United States | Yes (x2) |
| 4 | Indonesia |  |
| 5 | Pakistan | Yes |
| 6 | Nigeria |  |
| 7 | Brazil |  |
| 8 | Bangladesh |  |
| 9 | Russia |  |
| 10 | Ethiopia |  |
| 11 | Mexico | Yes |
| 12 | Japan | Yes |
| 13 | Egypt |  |
| 14 | Philippines | Yes |
| 15 | DR Congo |  |
| 16 | Vietnam |  |
| 17 | Iran |  |
| 18 | Turkey |  |
| 19 | Germany | Yes |
| 20 | Thailand |  |
| 21 | United Kingdom | Yes |
| 22 | Tanzania |  |
| 23 | France |  |
| 24 | South Africa |  |
| 25 | Italy | Yes |
| 26 | Kenya |  |
| 27 | Myanmar |  |
| 28 | Colombia |  |
| 29 | South Korea | Yes |
| 30 | Sudan |  |
| 31 | Uganda |  |
| 32 | Spain | Yes |
| 33 | Algeria |  |
| 34 | Iraq |  |
| 35 | Argentina | Yes |
| 36 | Afghanistan |  |
| 37 | Yemen |  |
| 38 | Canada |  |
| 39 | Poland |  |
| 40 | Morocco |  |
| 41 | Angola |  |
| 42 | Ukraine |  |
| 43 | Uzbekistan |  |
| 44 | Malaysia |  |
| 45 | Mozambique |  |
| 46 | Ghana |  |
| 47 | Peru |  |
| 48 | Saudi Arabia |  |
| 49 | Madagascar |  |
| 50 | Côte d'Ivoire |  |
| 51 | Nepal |  |
| 52 | Cameroon |  |
| 53 | Venezuela |  |
| 54 | Niger |  |
| 55 | Australia | Yes |
| 56 | North Korea |  |
| 57 | Syria |  |
| 58 | Mali |  |
| 59 | Burkina Faso |  |
| 60 | Taiwan |  |
| 61 | Sri Lanka |  |
| 62 | Malawi |  |
| 63 | Zambia |  |
| 64 | Kazakhstan |  |
| 65 | Chad |  |
| 66 | Chile |  |
| 67 | Romania |  |
| 68 | Somalia |  |
| 69 | Senegal |  |
| 70 | Guatemala |  |
| 71 | Netherlands | Yes |
| 72 | Ecuador |  |
| 73 | Cambodia |  |
| 74 | Zimbabwe |  |
| 75 | Guinea |  |
| 76 | Benin |  |
| 77 | Rwanda |  |
| 78 | Burundi |  |
| 79 | Bolivia |  |
| 80 | Tunisia |  |
| 81 | South Sudan |  |
| 82 | Haiti |  |
| 83 | Belgium |  |
| 84 | Jordan |  |
| 85 | Dominican Republic |  |
| 86 | United Arab Emirates |  |
| 87 | Cuba |  |
| 88 | Honduras |  |
| 89 | Czech Republic (Czechia) |  |
| 90 | Sweden | Yes |
| 91 | Tajikistan |  |
| 92 | Papua New Guinea |  |
| 93 | Portugal | Yes |
| 94 | Azerbaijan |  |
| 95 | Greece | Yes |
| 96 | Hungary |  |
| 97 | Togo |  |
| 98 | Israel |  |
| 99 | Austria |  |
| 100 | Belarus |  |
| 101 | Switzerland |  |
| 102 | Sierra Leone |  |
| 103 | Laos |  |
| 104 | Turkmenistan |  |
| 105 | Hong Kong |  |
| 106 | Libya |  |
| 107 | Kyrgyzstan |  |
| 108 | Paraguay |  |
| 109 | Nicaragua |  |
| 110 | Bulgaria |  |
| 111 | Serbia |  |
| 112 | El Salvador |  |
| 113 | Congo |  |
| 114 | Denmark | Yes |
| 115 | Singapore |  |
| 116 | Lebanon | Yes |
| 117 | Finland | Yes |
| 118 | Liberia |  |
| 119 | Norway | Yes |
| 120 | Slovakia |  |
| 121 | State of Palestine |  |
| 122 | Central African Republic |  |
| 123 | Oman |  |
| 124 | Ireland | Yes |
| 125 | New Zealand | Yes |

Taken from <https://www.worldometers.info/world-population/population-by-country/> (Accessed 12 February 2025)
